# There is no path to *ending AIDS* by 2030 without improving human rights

**DOI:** 10.1002/jia2.26197

**Published:** 2023-12-08

**Authors:** Stefan Baral, Gregorio Millett, Omar Syarif, Nguissali Turpin, Sheree Schwartz

**Affiliations:** ^1^ Center for Public Health and Human Rights Department of Epidemiology Johns Hopkins School of Public Health Baltimore Maryland USA; ^2^ Public Policy Office The Foundation for AIDS Research (amfAR) Washington District of Columbia USA; ^3^ Global Network of People Living with HIV (GNP+) Amsterdam Netherlands; ^4^ Enda Santé Dakar Senegal

1

2023 has witnessed worsening human rights contexts around the world with increasing conflict and violence, decreased legal protections and sustained or even increasing criminalization of historically marginalized communities. While the relationship between health and human rights has long been established, the evidence supporting this relationship available in 2023 is unequivocal in demonstrating that the fulfilment of human rights is central to improving the health of individuals and the communities of which they are a part [1]. The relationship between health and human rights has been established for many infectious and non‐communicable diseases but has been a defining characteristic of the HIV pandemic since its emergence over 40 years ago [2]. In any location or population where human rights are violated, HIV risks increase [3, 4]. And where human rights are fulfilled, the context to effectively deliver HIV prevention, testing and treatment programmes can be leveraged to decrease HIV incidence and improve the health and wellbeing of people living with HIV.

But in this fifth decade of the HIV response, there are some changing dynamics that reinforce the harms caused by suboptimal human rights contexts. First, the amount of resources available for people at high risk of acquiring or living with HIV is decreasing every day given fixed resources and growing communities of people living with HIV each year [5, 6]. Second, human rights contexts threaten to limit the potential positive impact of the ongoing switch to integrase strand transfer inhibitors (INSTIs) for the prevention and treatment of HIV. Third, worsening human rights contexts challenge the disclosure of historically stigmatized sexual and gender identities, occupations, substance use and dependency which challenge the planning and implementation of evidence‐based local HIV responses.

Several technical and normative agencies have established 2030 as the target date where HIV should no longer represent a major public health threat, assuming that all benchmarks are met [7]. However, the number of people living with HIV continues to grow due to both life‐saving treatment for HIV and new HIV acquisitions. Thus, there will soon be over 40 million people living with HIV, all in need of antiretroviral therapy (ART) to preserve life with a secondary benefit of U=U where people living with HIV who sustain viral suppression have no risk of onward HIV transmission. Moreover, millions of people around the world will continue to need access to different forms of pre‐exposure prophylaxis (PrEP) until the risk of HIV acquisition becomes negligible. It is increasingly clear that the level of resources for HIV will not increase given other threats to health including rapidly emergent infectious disease epidemics, the health effects of climate change, and the move towards integrated universal healthcare systems. Data from the People Living with HIV Stigma Index 2.0 collected from 2020 to 2022 have demonstrated sustained stigma and particularly high burden of stigma among youth [8]. In data from more than 10,000 cisgender women living with HIV from 11 countries across sub‐Saharan Africa, nearly 1 in 10 had experienced blackmail related to their HIV status, and this was associated with 35% decrease in adjusted odds of reporting current ART use (aOR 0.65, 95% CI 0.51–0.85) [9]. Moreover, young people living with HIV aged 18–24 were more likely to report enacted stigma in healthcare settings than people aged 25 and above [8]. Higher burdens of stigma affecting youth may be one of the critical drivers of both lower viral suppression rates among young people living with HIV and higher PrEP discontinuation rates among young women [10, 11, 12]. It simply is not possible to end HIV as a public health threat in successive generations when it is youth who are disproportionately at risk of HIV acquisition and experience suboptimal HIV outcomes if living with HIV [13].

Resistance mutations have long undermined commonly used first‐ and second‐line treatment regimens used in low‐ and middle‐income countries, resulting in sustained mortality among people living with HIV who were using treatment as prescribed. The switch to dolutegravir over the past few years as an INSTI is central to the plan to improve treatment outcomes given lower levels of resistance. However, historically, the prevalence of resistance mutations has not been evenly distributed among people living with HIV with higher burdens among marginalized communities, including sexual and gender diverse communities, sex workers, and people who use drugs [14]. Stigma drives intermittent care leading to the emergence of primary mutations and large sexual networks facilitate the propagation of these mutations. The barriers that, in part, drove the emergence of resistance remain present, potentially threatening the long‐term viability of dolutegravir as the core agent in first‐line treatment regimens. In addition, a major shift underway is the scale‐up of long‐acting injectables (LAIs) for HIV prevention and treatment of HIV. While LAIs hold great promise, the switch to LAIs does not negate the need for follow‐up even if that follow‐up is less regular. In HPTN‐083, which demonstrated the superiority of cabotegravir LAI (CAB‐LA) to daily oral PrEP, most of the acquisitions that happened were among people who were delayed in accessing their injections [15]. Moreover, nearly half of the people who acquired HIV after exposure to CAB‐LA developed INSTI resistance. Taken together, this suggests that unless individual, network and structural barriers to care including stigma are better understood and addressed, INSTI resistance may hasten with the scaling of LAIs.

The last decade has witnessed an improved understanding of the heterogeneity of risk even in broadly generalized HIV epidemics [16]. With increasing data collection, there has been a growing understanding that the same communities that have been known to be disproportionately affected since the emergence of HIV in higher income settings including gay men and other men who have sex with men, sex workers of all genders, transgender women, and people who use drugs were also at high risk in low‐ and middle‐income settings. A primary barrier to better understanding risk differentiation is limited disclosure of identity, secondary to attribute‐specific and intersectional stigmas and even the criminalization of people based on their consensual sexual preferences, occupations and substance dependencies. While there appeared to be some improvement in the respect, protection, and fulfilment of human rights secondary to the engagement and training of police, judges, journalists and religious leaders, these programmes have been mostly scaled down to increase resources for clinic‐based services [17]. Unlike scaling back treatment, the negative effects of scaling down services are not experienced immediately but take time to manifest. The recent intensification of the passing of punitive laws, negative media coverage and stigmatizing rhetoric has historically and will continue to decrease voluntary disclosure and ultimately the implementation of differentiated services to address HIV acquisition and transmission risks [18].

The harms of punitive policies and harsh social contexts have long been known and mentioned in countless studies, viewpoints and reports. But there is an evidence base supporting effective response strategies that mitigate these harms, such as stigma reduction interventions, person‐centred approaches to care and facilitating the equitable access to services. Yet, even in 2023, addressing structural determinants often remains rhetoric and is rarely centred in HIV responses.

There is no path to end AIDS by 2030 unless we as a global community do better.

## COMPETING INTERESTS

No competing interests were declared by the authors.

## AUTHORS’ CONTRIBUTIONS

SB developed the first draft of the manuscript with each co‐author providing conceptual inputs, critical review and edits.

## FUNDING

Effort for SB was supported by the National Institutes of Health (NIH) with the following awards (R01AI170249, R01MH132150, R01NR020437, R01MH110358) with effort for SS supported by R01MH121161.

## DISCLAIMER

The content is solely the responsibility of the authors and does not necessarily represent the official views of the National Institutes of Health.
